# Venous Thromboembolism Has a Variable Time of Occurrence in the Course of COVID-19: A Case Series

**DOI:** 10.7759/cureus.12295

**Published:** 2020-12-26

**Authors:** Tuoyo O Mene-Afejuku, Gini P Jeyashanmugaraja, Adedoyin A Akinlonu, Mohammed Osman, Mahfuz Hoq

**Affiliations:** 1 Epidemiology, Rollins School of Public Health, Emory University, Atlanta, USA; 2 Internal Medicine, Yale New Haven Health System/Bridgeport Hospital, Bridgeport, USA; 3 Radiology, Yale New Haven Health System/Bridgeport Hospital, Bridgeport, USA

**Keywords:** covid-19, sars-cov-2, venous thromboembolism (vte)

## Abstract

The novel severe acute respiratory syndrome coronavirus-2 (SARS-CoV-2) which results in coronavirus disease 2019 (COVID-19) has had a devastating impact globally. Not much is fully understood about this disease. Acute respiratory distress syndrome (ARDS) appears to be the commonest complication among patients with COVID-19. However, venous thromboembolism (VTE) appears to be a common complication among patients with COVID-19 even with adequate anticoagulation during hospitalization. VTE may confer a poor outcome on its own or may exacerbate other common complications such as ARDS or cardiac injury.

There are several diagnostic dilemmas with regards confirming VTE among patients with COVID-19 as there is a move to reduce the transfer of patients for angiographic studies or even venous Doppler ultrasonography because of the high transmissibility SARS-CoV-2. There is also the risk of worsening ARDS following fluid administration to prevent contrast nephropathy after angiographic studies.

It is, therefore, crucial to understand the timing of VTE occurrence in the setting of COVID-19, identify strategies for early diagnosis of VTE, therapeutic options as well as prognostic implications of VTE in the setting of COVID-19.

## Introduction

The world is grappling with the novel severe acute respiratory syndrome coronavirus-2 (SARS-CoV-2) which results in coronavirus disease 2019 (COVID-19) [1,2]. This disease has been noted to have varying associations and presentations [1,2]. The mortality rate associated with COVID-19 is variable, as data in this regard is constantly changing with each passing day. As of August 8th, 2020, there were more than 19 million confirmed cases of COVID-19 globally, of which there were 721,529 deaths [3]. Acute respiratory distress syndrome (ARDS) appears to be the commonest complication among patients with COVID-19 which in the report by Li et al. [4] occurred in about 38.3% of the study population. Cardiac injury was the second most common complication (21.7%) in the same study by Li et al. while diffuse intravascular coagulation occurred in 7.7% of the 548 patients studied.

Venous thromboembolism (VTE) has been reported in a few reports of patients with COVID-19 [5,6]. There is a lot of diagnostic dilemma with regards to confirming VTE among patients with COVID-19 as there is a move to reduce the transfer of patients for angiographic studies or even venous Doppler ultrasonography because of the high transmissibility SARS-CoV-2 [5-7]. There is also the risk of worsening ARDS following fluid administration to prevent contrast nephropathy after angiographic studies as well as the possibility that patients may be too unstable to carry out angiographic studies. The increased level of D-dimer in association with COVID-19 has also heightened the difficulty in ascertaining the presence or predicting the occurrence of VTE [4-7]. As a result, there seem to be more questions than answers with regards to the relationship between D-dimer and VTE, strategies for early identification of thromboembolic complications, therapeutic options, and prognostic implications of VTE in the setting of COVID-19.

We, therefore, present this case series in an attempt to elucidate on VTE as a crucial complication of COVID-19 and provide areas of further exploration which may help in improving outcomes among affected patients.

## Case presentation

The first two cases presented here were individuals who had a relatively benign course of COVID-19 who were readmitted because of features in keeping VTE despite adequate prophylaxis during the index admission. The last two cases had VTE during the index admission for COVID-19 and were markedly older than the first two cases.

Case 1

A 67-year-old African American man with a history of type 2 diabetes mellitus, hypertension, obesity, and hyperlipidemia presented at Bridgeport hospital on account of a two-day history of low-grade fever (100.6F), headache, and polyuria. He was also noted to be short of breath (SOB) at presentation (oxygen saturation of 95% on room air) and a heart rate of 101/min. His blood pressure was elevated on examination and he was morbidly obese with a body mass index of 42kg/m^2^. He tested positive for SARS-CoV-2. Laboratory findings were notable for elevated inflammatory markers such as white cell count (WBC), lactate dehydrogenase (LDH), C-reactive protein (CRP), and ferritin as shown in Table 1. He also had elevated D-dimer (2.47 mg/L FEU) which is one of the routine testing done for patients with COVID-19. Computerized tomography pulmonary angiogram (CT-PA) was not done because of elevated serum creatinine of 1.44mg/dl as well as the decreased role of CT-PA during the early phase of the COVID-19 pandemic [5-7]. His pretest probability for VTE was low with a Well’s score of 2 (prolonged immobilization and tachycardia). He got prophylaxis for VTE with subcutaneous heparin 5000 units three times a day (TID). Chest X-ray (CXR) was unremarkable. He received hydroxychloroquine 200 mg twice a day (BID) for 10 days as therapy for COVID-19 as per the protocol. He also received supplemental oxygen via nasal cannula for most of the time during his 12-day hospital stay. This was titrated down until he was off oxygen and symptom-free before discharge home.

**Table 1:** Case presentation summary Ref= reference range; LDH= lactate dehydrogenase.

	Case 1	Case 2	Case 3	Case 4
Age(years)	67	58	89	82
Gender	male	female	female	female
Race	African American	Hispanic	White	African American
Co-morbidities	Hypertension, type 2 diabetes mellitus, obesity, and hyperlipidemia	hypothyroidism	Hypertension, Hyperlipidemia, Dementia, and Hypothyroidism	Hypertension, type 2 diabetes mellitus, and obstructive sleep apnea
Clinical Presentation				
Symptoms	Index admission Fever, headache, and polyuria Readmission Shortness of breath and low oxygen saturation	Index admission Fatigue and cough Readmission Worsening shortness of breath since discharge	Fever, Shortness of breath	Cough, Shortness of breath
Duration of symptoms	Index admission 2 days Readmission 2 days	Index admission 3 days Readmission 8 days	1 day	2 days
Physical examination				
Temperature	Index admission 100.6F, Readmission 98.6F	Index admission 98 F Readmission 96 F	101.8F	97.6F
Oxygen saturation	Index admission 95% Readmission 93%	Index admission 98% Readmission 89%	88%	88%
Heart rate	Index admission 101/min Readmission 110/min	Index admission 92 /min Readmission 117/min	85/min	77/min
Respiratory rate	Index admission 20/min Readmission 20/min	Index admission 18 /min Readmission 36/min	30/min	18/min
Blood pressure	Index admission 162/90mmHg Readmission 144/90mmHg	Index admission 125/61mmHg Readmission 133/80mmHg	158/78mmHg	196/74mmHg
Well’s score	Index admission 2 Readmission 6	Index admission 0 Readmission 6	3	0
PERC score	Index admission 2 Readmission 3	Index admission 1 Readmission 3	2	2
Laboratory findings				
White cell count (ref: 4000-10000 /µL)	Index admission 10,900/µL Readmission 10,300/µL	Index admission 5,800/µL Readmission 12,100/µL	10,800/µL	8,000/µL
Lymphocyte Count (ref: 500- 5400/µL)	Index admission 2,420/µL Readmission 2,300/µL	Index admission 609/µL Readmission 1550/µL	4,000/µL	1,900/µL
Platelet count (ref: 120,000-450,000 /µL)	Index admission 176,000/µL Readmission 567,000/µL	Index admission 190,000/µL Readmission 214,000/µL	206,000/µL	346,000/µL
LDH (ref: 122-241 U/L)	Index admission 261 Readmission 380	Index admission 238 Readmission -	-	-
CRP, high sensitivity (ref: >10.0 is associated with infection and inflammation)	Index admission 19.9 Readmission 6.7	Index admission 41.3 Readmission 55.2	101.2	19.1
Ferritin (ref: 30-400 ng/ml)	Index admission 717ng/ml Readmission 1515ng/ml	Index admission 253ng/ml Readmission 470 ng/ml	255ng/ml	521ng/ml
Procalcitonin (ref: ≤ 0.25 ng/ml)	Index admission 0.15 Readmission 0.06	Index admission 0.10 Readmission 0.14	0.46	0.11
D-dimer (ref:<=0.67 mg/L FEU)	Index admission 2.47 mg/L FEU Readmission 8.57 mg/L FEU	Index admission 0.41 mg/L FEU Readmission >35.2 mg/L FEU	>35.2 mg/L FEU	35.2 mg/L FEU
Serum creatinine (ref: 0.40- 1.30 mg/dl	Index admission 1.44 mg/dl Readmission 1.27 mg/dl	Index admission 0.9 mg/dl Readmission 0.91 mg/dl	1.05 mg/dl	1.38 mg/dl
QTC interval	Index admission 423ms Readmission 491ms	Index admission 445 ms Readmission 463 ms	472 ms	461 ms
Chest x-ray	Index admission Normal Readmission bilateral airspace opacities and interstitial prominence	Index admission Left basal consolidation. Readmission patchy left lung base infiltrate with subtle infiltrative changes in the right upper lobe	normal	Bilateral airspace opacities
Computerized chest tomographic angiogram	Readmission Multiple filling defects within bilateral lower and left upper segmental pulmonary artery branches.	Readmission Large saddle embolus from the main pulmonary artery into right and left pulmonary arteries extending to lobar, segmental, and subsegmental pulmonary arteries of all lobes bilaterally	Bilateral pulmonary embolism with mild right heart strain	Bilateral acute pulmonary artery embolism
Therapy	Index admission hydroxychloroquine Readmission Anticoagulation with apixaban	Index admission hydroxychloroquine Readmission Anticoagulation with low molecular weight heparin and apixaban	hydroxychloroquine , ceftriaxone, doxycycline, heparin drip, and then apixaban	hydroxychloroquine low moloecular weight heparin and then apixaban
Type of oxygen therapy	Index admission Nasal cannula Readmission Nasal cannula	Index admission None Readmission nasal cannula	Nasal cannula	Nasal cannula
Length of hospital stay	Index admission 12 days Readmission 7 days	Index admission 4 days Readmission 8 days	19 days	4 days
Outcome	Discharged home	Discharged home	Nursing home	Discharged home
Time to readmission	2 days	8 days	-	-

**Figure 1:**
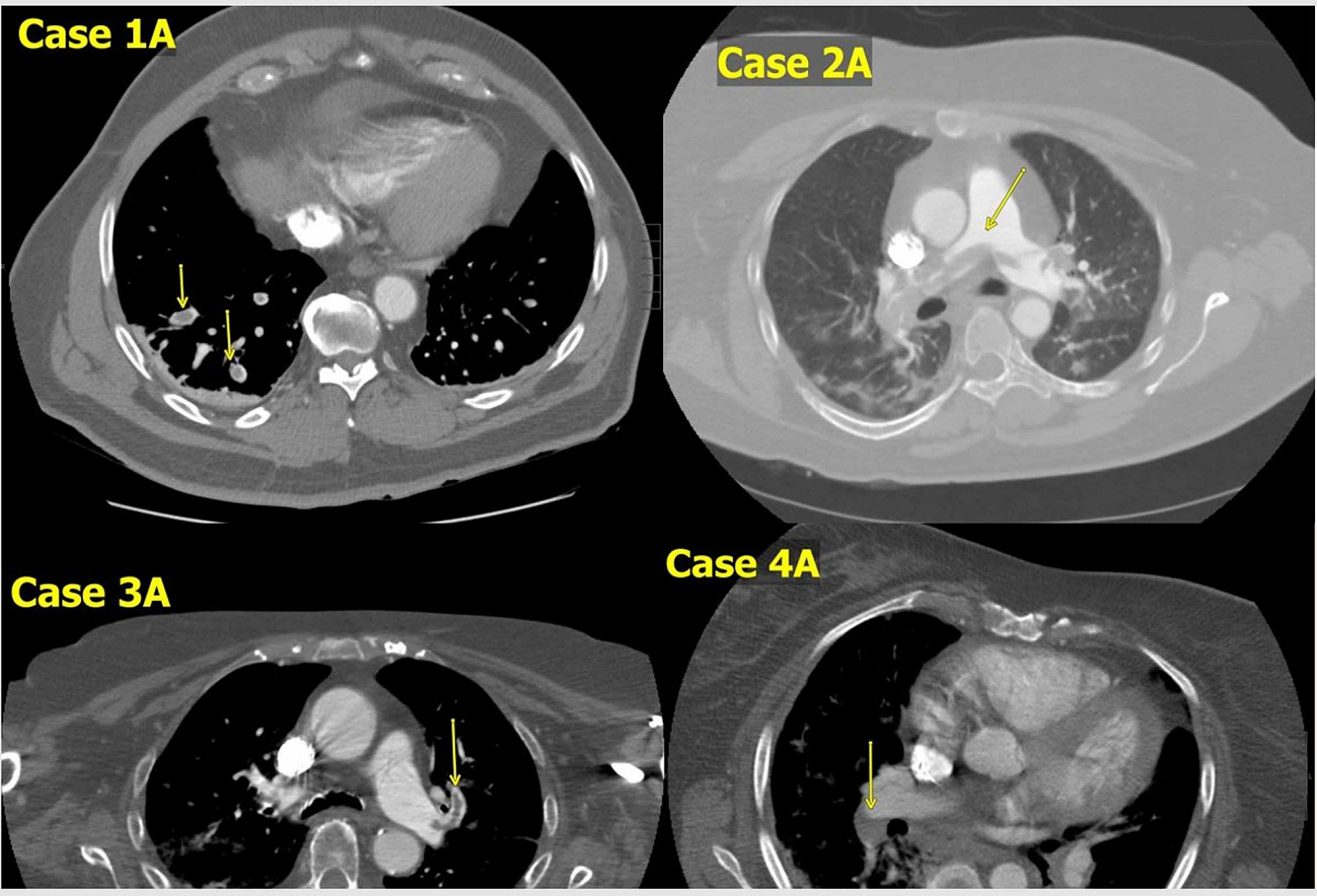
Computerized tomographic chest angiograms of the patients in this case series. Case 1A: Segmental pulmonary embolus. Case 2A: Pulmonary artery saddle embolus. Case 3A: Segmental branching embolus. Case 4A: Right lower lobe segmental embolus.

However, two days after discharge, he was readmitted because of sudden onset SOB and low oxygen saturation (93% on room air) noted by his home health aide. D-dimer this time around was higher than at presentation (8.57 mg/L FEU). Chest x-ray now revealed bilateral airspace opacities and interstitial prominence. He had CT-PA which revealed multiple filling defects within bilateral lower and left upper segmental pulmonary artery branches as shown in Figure 1. He was anticoagulated with apixaban 10 mg BID for seven days and received intranasal oxygen via nasal cannula. He was discharged home on apixaban 5 mg BID.

Case 2

A 58-year-old Hispanic female with a history of hypothyroidism presented on account of a three-day history of non-productive cough and fatigue. Her vital signs and physical examination findings were unremarkable as shown in Table 1. Laboratory findings were remarkable for positive SARS-CoV-2 and elevated CRP (41.3). Other inflammatory markers were normal as well as normal procalcitonin and D-dimer (Table 1). Her CXR however revealed left basal consolidation. She did not require supplemental oxygen and was discharged home after a hospital stay of four days. She was not on antibiotics even with the consolidation on CXR because this was thought to be due to SARS-CoV-2 and not bacterial pneumonia as white cell count and procalcitonin were within normal limits. She got the customary prophylaxis for VTE with subcutaneous heparin 5000 units TID.

Eight days after her discharge home, she developed SOB which worsened progressively. She had tachycardia, tachypnea, and low oxygen saturation of 89% on readmission. Inflammatory markers were higher than at the initial admission but procalcitonin remained normal (Table 1). Well’s score was 6 compared to zero at the initial admission. Worsening of the infiltrates was also noted on chest X-ray compared to that on admission. D-dimer was elevated (>35.2 mg/L FEU ) and CT-PA revealed a large saddle embolus from the main pulmonary artery into right and left pulmonary arteries extending to lobar, segmental, and subsegmental pulmonary arteries of all lobes bilaterally as shown in Figure 1.

A focused cardiac ultrasound was done which revealed moderate to severe right ventricular dilation. Due to hemodynamic stability and unclear mortality benefit of catheter directed thrombolysis , a decision was made to pursue anticoagulation with low molecular weight heparin (1 mg/kg BID). She received supplemental oxygen via nasal cannula and discharged home on apixaban.

Case 3

An 89-year-old Caucasian female with a history of hypertension, hyperlipidemia, hypothyroidism, and dementia presented on account of fever (101.8F) and SOB for one day. She had tachypnea and low oxygen saturation of 88%. CRP and D-dimer were elevated at 101.2 and >35.2 mg/L FEU respectively. She also had mild leukocytosis (10,800/µL). CXR was unremarkable but CT-PA revealed bilateral pulmonary embolism with mild right heart strain as shown in Figure 1. The patient received hydroxychloroquine, ceftriaxone, doxycycline, and heparin drip. She was initially treated with parenteral high molecular weight heparin with loading dose of 80 units/kg bolus followed by maintanence dosing at 18 units/kg/hr. She received supplemental oxygen via nasal cannula and spent a total of 19 days before she was discharged to a nursing home on apixaban 5 mg BID .

Case 4

An 82-year-old African American female with a history of hypertension, type 2 diabetes mellitus, and obstructive sleep apnea presented on account of cough and SOB of two days duration. She was afebrile but had a low oxygen saturation of 88%. Her blood pressure was markedly elevated (196/74mmHg). CRP and ferritin were mildly elevated as shown in Table 1. She also had markedly elevated D-dimer (35.2 mg/L FEU). The CXR revealed bilateral airspace opacities. She was treated empirically with low molecular weight heparin 1 mg/kg BID and hydroxychloroquine 200 mg BID. CT-PA done two days after admission revealed bilateral acute pulmonary artery embolism as shown in Figure 1. She received supplemental oxygen and was discharged home after a four-day hospital stay on apixaban 5 mg BID. 

## Discussion

VTE appears to be one of the emerging and critical complications of COVID-19 which may have far-reaching effects in terms of morbidity and mortality [1-4]. It may confer its deleterious effects singly or in conjunction with other sinister complications such as ARDS and myocardial injury [1-4]. Another concern about the occurrence of VTE among patients with COVID-19 is that the timing of its occurrence is variable as it may occur prior to hospitalization, on admission (cases 3 and 4) and upon discharge (cases 1 and 2). It is important to note that this timing is directly related to the duration and severity of the disease.

Most reports indicate adverse events are commoner among elderly patients whose risks may be compounded by co-morbidities [4,7]. Younger individuals and others with a low pretest probability of VTE appear to still be a risk of VTE in the setting of COVID-19 as seen in the case descriptions above in Table 1. There appear to be several plausible reasons for the occurrence of VTE among patients with COVID-19. This may be due in part to the association of SARS-CoV-2 infection with lymphopenia (case 2 with lymphocyte count less than 1000/µL), elevated lactate dehydrogenase, C-reactive protein, D-dimer, ferritin, and interleukin-6 (IL-6) [7,8]. These inflammatory markers, especially IL-6, have been noted to be indicators of severity and hypercoagulability state [7-9].

The four cases in this series had elevated levels of all the above markers to variable degrees. They all had elevated D-dimer which was extremely high (>35) among the individuals who had pulmonary embolism detected while on admission (cases 3 and 4) compared to relatively lower but elevated values in patients who were readmitted because of VTE (cases 1 and 2). The D-dimer was also noted to be very high on readmission in case 2 (>35) and moderately high in case 1 when compared to values at the index admission. This may imply that very high levels of D-dimer may correlate with a high risk of the presence of VTE as described by other authors [7-9]. It is, however, a well-known fact that D-dimer is mainly useful as a negative predictor of VTE and as a result, positive results may not be really meaningful [10] unless the pretest probability of VTE is very high. The differential timing of the occurrence of VTE in this case series may also imply that other markers of inflammation and hypercoagulation independently or in conjunction with D-dimer may need to be investigated to have a more robust predictive assessment of VTE events. 

All patients in this series were on adequate prophylactic medications for VTE while on admission but still developed pulmonary embolism at some point in the course of the disease as shown in Figure 1 [11]. The suspicion of VTE in these two cases was based on remarkably higher D-dimer in comparison to that on admission. It is important to note that a high index suspicion was required to make the diagnosis of pulmonary embolism as the pretest probability of both patients (cases 1 and 2) at the index admission for VTE was low. Screening for deep vein thrombosis with venous Doppler ultrasound on the proposed day of discharge among patients treated for COVID-19 may be a useful strategy in preventing re-hospitalization due to VTE. It is unclear if a diagnosis of COVID-19 necessitates prophylactic oral anticoagulation therapy upon discharge. Further studies would be required to risk-stratify patients with COVID-19 with regards to their candidacy for short term prophylactic anticoagulation therapy upon discharge. The length of hospital stay, age of the patient, co-morbid states, need for care in the intensive care unit, may all play a role in the determination of an individual’s risk of VTE in the setting of COVID-19. Further exploration would be required in this regard.

**Figure 2:**
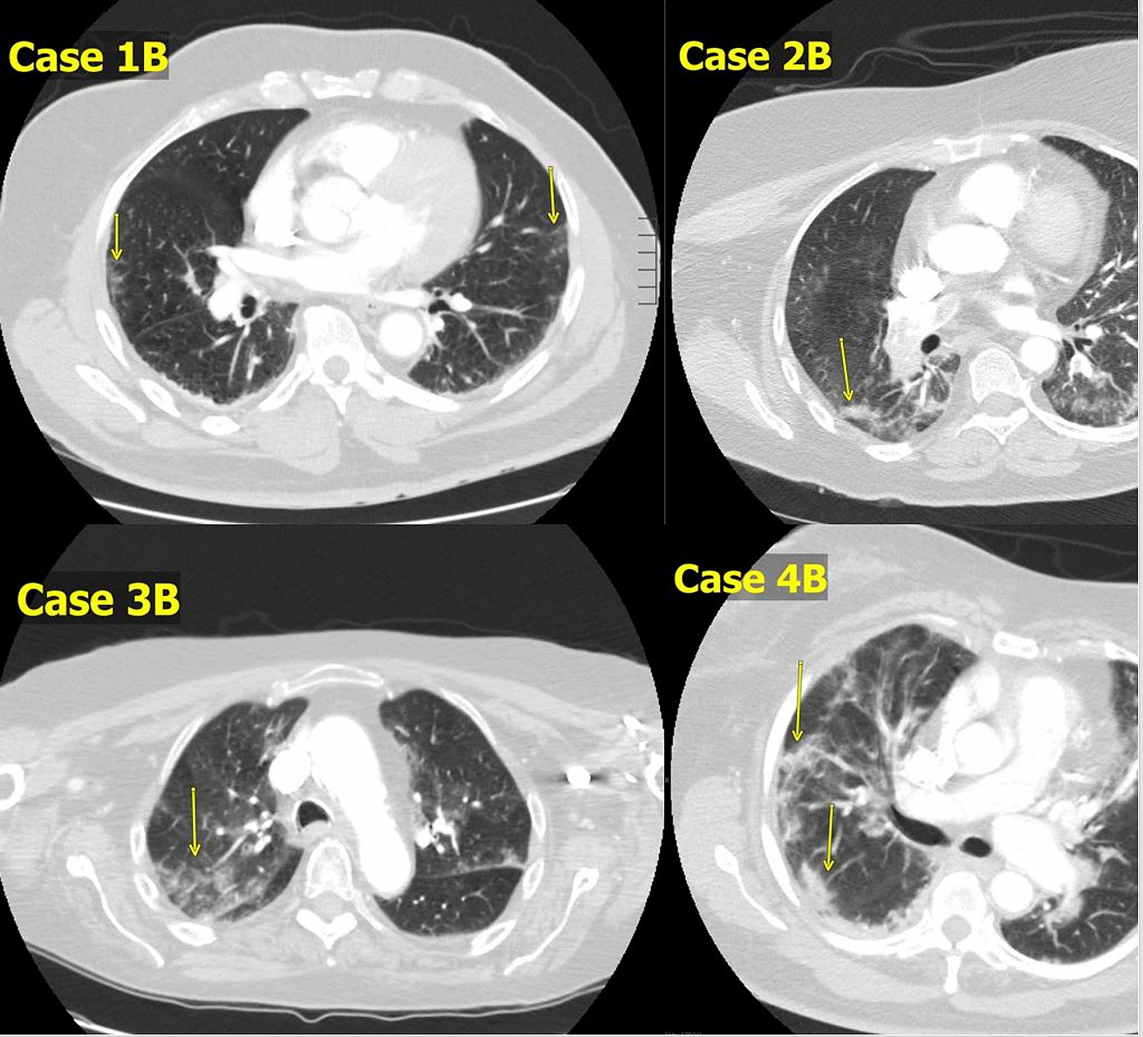
Computerized tomography of the chest of the patients in this case series. Case 1B: patchy peripheral ground-glass opacity. Case 2B: patchy peripheral ground-glass opacity. Case 3B: patchy peripheral ground glass opacity. Case 4B: patchy peripheral ground glass opacity

On the whole, a high index of suspicion for VTE should be entertained in the setting of COVID-19 if hypoxemia is disproportionate to the degree of known respiratory pathologies (ground-glass opacities and other evidence of lung damage as shown in Figure 2), or if it occurs acutely in the setting of unexplained right ventricular dysfunction [7].

## Conclusions

VTE occurs at variable time periods in the course of COVID-19. Its occurrence is difficult to predict, and a high index of suspicion is required to institute early therapy and most likely improve outcomes.
